# Hip pain - an uncommon cause of extensive deep vein thrombosis (DVT) in the paediatric age group: a case report

**DOI:** 10.11604/pamj.2025.50.69.46977

**Published:** 2025-03-11

**Authors:** Adebayo DaCosta, Suprina Gurung, Ayokunle Osonuga, Demilade DaCosta, Moyoninuoluwa Adegbite

**Affiliations:** 1 Department of Emergency Medicine, Medway Maritime Hospital, Gillingham, Kent, United Kingdom; 2 Department of General Practice, Coltishall Medical Practice, Coltishall, Norfolk, United Kingdom; 3 Department of Primary Care, Norwich Medical School, University of East Anglia, Norwich, United Kingdom; 4 Faculty of Life Sciences and Medicine, Kings College London, London, United Kingdom; 5 Faculty of Medicine and Health Sciences, University of Buckingham Medical School, Buckingham, United Kingdom

**Keywords:** Deep vein thrombosis, anticoagulants, paediatrics, case report

## Abstract

Deep vein thrombosis (DVT) in the pediatric population remains an infrequent yet clinically significant entity, with an estimated incidence of 0.07-0.14 per 10,000 children. We describe a unique case involving a previously healthy 16-year-old female who presented with progressively worsening left hip pain radiating to the knee, inability to weight bear, and associated limb heaviness with a purple discoloration on standing. Clinical evaluation revealed unilateral leg swelling, increased girth, and pain on passive joint movement, while initial radiographs were unremarkable; however, a markedly elevated D-dimer level prompted further investigation with Doppler ultrasound, which confirmed an extensive iliofemoral DVT. Owing to the lack of pediatric-specific guidelines, management was adapted from adult protocols, including thrombolytic therapy with tissue plasminogen activator and subsequent anticoagulation with dalteparin and apixaban, despite a complication of epistaxis that influenced the treatment course. This case highlights the diagnostic challenges inherent in pediatric DVT, highlights the necessity for a high index of suspicion when evaluating hip or leg pain in children, and emphasizes the urgent need for the development of dedicated pediatric guidelines to inform optimal therapeutic strategies.

## Introduction

Deep vein thrombosis (DVT) in children is a rare event, with an incidence estimated between 0.07 and 0.14 per 10,000, and is most commonly associated with underlying risk factors such as congenital heart disease, malignancy, or trauma [1,2]. The diagnostic process is further complicated by the absence of validated pediatric venothromboembolic scoring systems, often resulting in delayed recognition when symptoms are subtle or atypical. Moreover, current management strategies are largely extrapolated from adult protocols, reflecting a significant gap in pediatric-specific evidence-based guidelines [3,4].

We present the case of a previously healthy 16-year-old female who developed an extensive iliofemoral DVT, initially manifesting as isolated hip pain- a presentation that deviates markedly from the typical musculoskeletal etiologies encountered in this age group. The atypical clinical presentation and the ensuing diagnostic and therapeutic challenges highlight the need for heightened clinical vigilance and the development of tailored management guidelines for pediatric thromboembolic events. This report aims to enrich the limited body of literature on pediatric DVT and to stimulate further research into optimizing diagnostic and treatment strategies for this vulnerable population.

## Patient and observation

**Patient information:** a previously healthy 16-year-old female presented to the emergency department accompanied by her mother. The primary complaint was a progressively worsening left hip pain over a three-day period, which subsequently extended to the left knee, resulting in an inability to weight bear. The patient described the affected limb as feeling heavy, with a noted purple discoloration upon standing. She had no significant past medical history and was not taking any medications (including oral contraceptives), and there was no contributory family or genetic history. No previous similar episodes or interventions were reported.

**Clinical findings:** on physical examination, the left leg exhibited a mottled appearance compared to the right, with notably tight, tense, and rigid skin and musculature extending from the thigh to the calf. Objective measurements revealed an asymmetry with the left thigh measuring 43.6 cm versus 40.5 cm on the right and the left calf measuring 33.8 cm compared to 32.7 cm on the right. There was pain elicited on passive flexion and extension at the hip joint, although palpation of the long bones and bony prominences did not reproduce discomfort.

### Timeline of current episode

**Figure 1:**
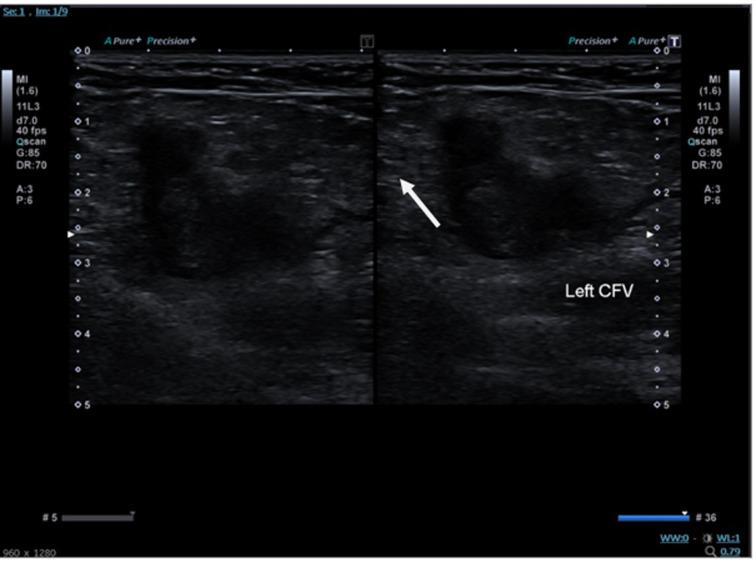
Doppler ultrasound slide showing thrombus (white arrow) in the left common femoral vein

Day 1-3: onset of left hip pain, progressively worsening and extending to the left knee, culminating in an inability to weight bear. Day 3 (presentation): the patient presented to the emergency department; initial evaluation and pelvic/hip radiographs were performed, which revealed no bony abnormalities. Emergency department (ED) evaluation: a significantly elevated D-dimer (9832 ng/mL; reference range 0.0-243.0 ng/mL) prompted further investigation. Admission: the patient was admitted under the pediatric team for further evaluation. Diagnostic imaging: urgent Doppler ultrasound imaging confirmed an extensive iliofemoral deep vein thrombosis (Figure 1).

**Intervention:** thrombolysis was initiated via placement of a 50 cm infusion catheter with a tPA regimen (2 mg/hr for 4 hours followed by 1 mg/hr), and a subsequent venogram demonstrated revascularization with residual thrombus. A transient episode of severe epistaxis precluded stent placement, leading to a modified anticoagulation strategy.

**Post-intervention:** the patient was managed with dalteparin (10,000 mcg) and apixaban (10 mg twice daily for one week, followed by 5 mg twice daily for three months), with scheduled follow-up in the pediatric hematology clinic and ultrasound surveillance.

**Diagnostic assessment:** initial diagnostic imaging with pelvic and left hip X-rays was unremarkable, ruling out bony pathology. The markedly elevated D-dimer, in conjunction with clinical findings, raised suspicion for thromboembolic disease. Confirmation was achieved with a Doppler ultrasound study (Figure 1), which identified extensive thrombus formation in the deep venous system, consistent with an iliofemoral DVT. The diagnostic process was challenged by the absence of validated pediatric venothromboembolic scoring systems, necessitating reliance on protocols extrapolated from adult populations.

**Diagnosis:** the final diagnosis was a left iliofemoral deep vein thrombosis. Differential considerations initially included musculoskeletal etiologies and other causes of hip pain; however, the clinical presentation and diagnostic findings substantiated a thromboembolic event. The prognosis was deemed favorable following prompt and appropriate intervention.

**Figure 2:**
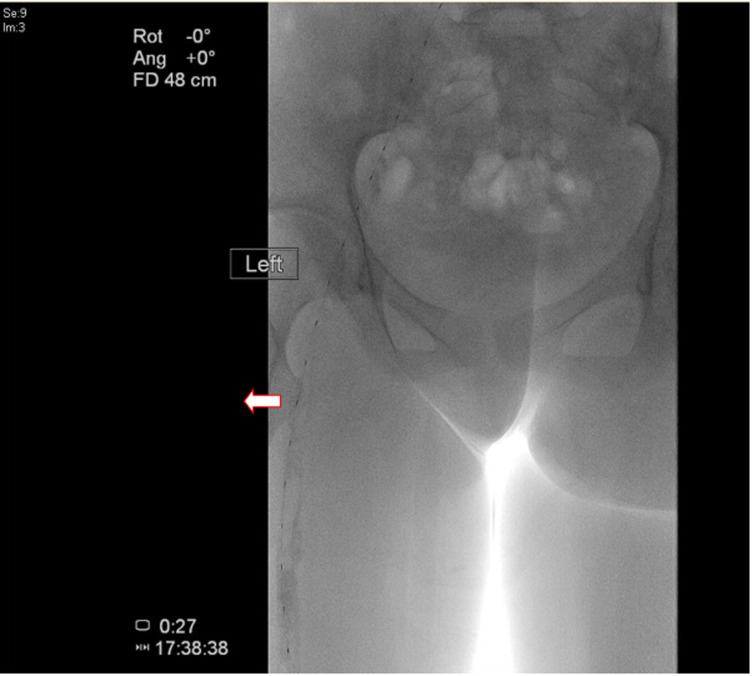
post thrombolysis showing a significant amount of residual thrombus in the (left) iliac and femoral veins

**Therapeutic interventions:** the patient underwent thrombolytic therapy with placement of a 50 cm infusion catheter across the thrombus, administering tPA at 2 mg/hr for four hours followed by 1 mg/hr. A post-thrombolysis venogram confirmed revascularization but also revealed a substantial residual thrombus (Figure 2). Due to the onset of severe epistaxis during the procedure, stenting was deferred. The hematology team subsequently initiated anticoagulation therapy with dalteparin (10,000 mcg) and apixaban (10 mg twice daily for one week, then 5 mg twice daily for three months), with the patient being closely monitored throughout her hospital stay.

**Follow-up and outcome of interventions:** the patient demonstrated remarkable clinical improvement with the resolution of acute symptoms and progressive mobilization during hospitalization. She was discharged after five days on a regimen of oral anticoagulants with planned follow-up in the pediatric hematology clinic and scheduled ultrasound surveillance to monitor thrombus resolution and venous patency. Adherence to the therapeutic regimen was ensured through regular clinical assessments, and the transient adverse event (epistaxis) was managed effectively without long-term sequelae.

**Patient perspective:** the patient reported a significant reduction in pain and an overall sense of relief following the initiation of treatment. She expressed gratitude for the multidisciplinary approach that not only addressed her acute symptoms but also provided reassurance through scheduled follow-up and continuous support. Her perspective underscored the importance of clear communication and prompt intervention in alleviating both the physical and psychological burdens of her condition.

**Informed consent:** consent for the publication of this case report was obtained from the patient and her legal guardian after a thorough explanation of the treatment, its outcomes, and the intent to contribute to the scientific literature on pediatric deep vein thrombosis.

## Discussion

The incidence of deep vein thrombosis (DVT) in children is notably low, estimated at 0.07 to 0.14 per 10,000, yet its clinical consequences can be severe, particularly when diagnosis is delayed or missed [1]. Unlike adults, pediatric patients often present with atypical symptoms and lack the classic risk factors such as advanced age or significant comorbidities, making early recognition inherently challenging. This case called the attention of clinicians to the necessity for heightened clinical vigilance, as even subtle presentations may herald extensive thrombotic disease.

A major challenge in pediatric DVT lies in the limitations of diagnostic modalities and the absence of validated pediatric-specific scoring systems. Tools such as D-dimer assays and compression ultrasonography, while robust in adult populations, exhibit variable sensitivity and specificity in children [5,6]. Consequently, a thorough clinical evaluation supported by imaging remains the cornerstone of diagnosis. The current reliance on adult-derived protocols emphasizes a critical gap in pediatric research and the urgent need for dedicated studies to refine diagnostic criteria for this vulnerable population.

Therapeutic management in pediatric DVT is equally complex. Current treatment regimens- primarily involving low molecular weight heparin (LMWH) and vitamin K antagonists are largely extrapolated from adult data, despite the distinct hemostatic physiology observed in children [4,7].

While catheter-directed thrombolysis and mechanical thrombectomy have emerged as promising options for extensive or limb-threatening thromboses, robust pediatric evidence remains scarce, and these interventions carry inherent risks, including bleeding complications. This case highlights the necessity for a multidisciplinary approach, integrating expertise from emergency medicine, radiology, and paediatric hematology to tailor therapy to the individual patient [8].

The strengths of this report include a comprehensive documentation of the clinical presentation, a meticulous diagnostic workup, and a clearly articulated therapeutic strategy. It serves as a valuable addition to the limited literature on pediatric DVT by illustrating both the potential severity of the condition and the limitations of current diagnostic and treatment paradigms. However, as a single case report, its findings are not generalizable, and the reliance on adult-based protocols suggests the need for future multicenter studies and randomized controlled trials specifically addressing pediatric thromboembolism.

## Conclusion

In summary, this case exemplifies the complexities inherent in the diagnosis and management of paediatric DVT. It reinforces the need for improved, evidence-based guidelines tailored to the unique physiological and clinical context of children, which will ultimately enhance patient outcomes and reduce long-term complications such as post-thrombotic syndrome.
